# Supplementary material for: Hybrid-hybrid correction of errors in long reads with HERO
Source: Genome Biol. 2023 Dec 1;24:275. doi: 10.1186/s13059-023-03112-7 (PMC10690975; doi:10.1186/s13059-023-03112-7)
Supplement: Supplementary file 1 — Additional file 1. Supplement. This contains all supplementary materials referenced in the main text. [file 13059_2023_3112_MOESM1_ESM.pdf]

Supplementary Material
Hybrid-hybrid correction of errors in long reads with HERO

Xiongbin Kang[1, 2], Jialu Xu[1], Xiao Luo[1,2,*], Alexander Schönhuth[2,*]

[1] College of Biology, Hunan University, Changsha, China
[2] Genome Data Science, Faculty of Technology, Bielefeld University, Bielefeld, Germany

*To whom correspondence should be addressed.

Supplementary Methods

Commands and versions of tools used for comparison.

Ratatosk 0.7.6
Ratatosk correct -s short_reads.fastq -l long_reads.fastq -o output_file

FMLRC2 0.1.8
cat short_reads.fastq | awk 'NR % 4 == 2' | sort | tr NT TN | ropebwt2 -LR | tr NT TN | fmlrc2-convert comp_msbwt.npy
fmlrc2 comp_msbwt.npy long_reads.fastq output_file

LoRDEC v0.9
lordec-correct -k 21 -s 5 -2 short_reads.fastq -i long_reads.fastq -o output_file

Canu v2.1.1
Assemble metagenomic sequencing data

canu genomeSize=genomesize -nanopore raw_long_read
canu genomeSize=genomesize -pacbio raw_long_read
canu genomeSize=genomesize -pacbio-hifi corrected_long_read

Hifiasm_meta v0.13-r308
hifiasm_meta -o output_file corrected_long_read

Assemble diploid/polyploid sequencing data

Arabidopsis thaliana

canu -assemble genomeSize=genomesize -corrected -pacbio corrected_long_read

Others diploid/polyploid sequencing data

canu -assemble genomeSize=genomesize stopOnLowCoverage=3 minInputCoverage=3 -corrected -nanopore corrected_long_read

canu -assemble genomeSize=genomesize stopOnLowCoverage=3 minInputCoverage=3 -corrected -pacbio corrected_long_read

BFC vr181
bfc -s genomesize short_reads.fastq >corrected_short_reads.fastq

Fastp v0.19.11
fastp -i long_reads.fastq -Q -l 1000 -o filter_long_reads.fastq

Table S1. GenBank numbers of reference genomes, and sample information and assembly results of real gut metagenome sequencing data.
| Additional file 1: Table S1.xlsx |

Methods	CPU Time (h)	Peak Memory Usage (GB)
FMLRC	**0.29**	**0.35**
HERO	1.54	1.00
Ratatosk	1.79	0.51
LoRDEC	2.40	1.84

Table S2. Runtime and memory usage for correcting simulated PacBio CLR reads of 3 *Salmonella* strains. The coverage of short and long reads both is 10X

.

Genomes	GenBank no	Coverage	ANI (%)
Streptococcus_thermophilus_isolate_NWC_1_1	CP029252.1	56.29	99.99
Streptococcus_thermophilus_isolate_NWC_2_1	CP031021.1	55.07	
Lactobacillus_delbrueckii_subsp.lactis_isolate_NWC_1_2	CP029250.1	39.38	99.24
Lactobacillus_delbrueckii_subsp.lactis_isolate_NWC_2_2	CP031023.1	35.13	
Lactobacillus_helveticus_isolate_NWC_2_4	CP031018.1	17.59	98.03
Lactobacillus_helveticus_isolate_NWC_2_3	CP031016.1	10.27	

Table S3. Average nucleotide identity (ANI) and coverage of Illumina reads for the 6 strains in the NWc data set.

Correction	Indels/100 kbp	Mismatches/100 kbp	GF(%)	MC(%)	N/100 kbp
Bmock12 ONT					
3Ratatosk	35.41	58.15	93.75	2.58	0.99
3Ratatosk_Racon	11.79	169.42	93.77	2.60	0.04
3Ratatosk_HERO	**9.80**	**42.50**	93.77	2.58	0.24
Bmock12 PacBio					
3Ratatosk	369.30	139.52	91.71	10.38	1.66
3Ratatosk_Racon	285.39	243.38	92.04	10.96	0.12
3Ratatosk_HERO	**197.70**	**98.12**	92.01	11.43	0.28
NWCs ONT					
3Ratatosk	489.02	621.13	99.99	7.06	26.14
3Ratatosk_Racon	288.95	548.67	100.00	7.23	7.94
3Ratatosk_HERO	**245.68**	**457.34**	100.00	7.37	14.56
NWCs PacBio					
3Ratatosk	360.58	422.17	83.12	6.12	13.65
3Ratatosk_Racon	335.37	409.34	83.65	5.82	5.05
3Ratatosk_HERO	**276.42**	**365.52**	84.22	5.73	9.93

Table S4. Here we compare the correction effects of different overlap-graph corrections. The first row in each subtable shows the error rates after 3 rounds of correction using Ratatosk. The second row in each subtable represents the error rates after 3 rounds of Ratatosk correction followed by one round of Racon correction. The third row in each subtable shows the error rates after 3 rounds of Ratatosk correction and one round of HERO correction.

Assembly	GF(%)	Indels/100 kbp	Mismatches/100 kbp	NGA50	N/100 kbp	MC(%)
Bmock12 ONT						
Canu	57.13	504.95	140.75	78498	0.00	9.04
Canu_Racon	57.15	8.29	**145.97**	78498	0.02	9.04
Canu_HERO	57.46	11.79	73.22	78498	0.04	9.04
Bmock12 PacBio						
Canu	50.01	174.71	63.79	6025	0.00	3.72
Canu_Racon	50.01	4.32	**104.21**	6025	0.00	3.72
Canu_HERO	50.01	4.30	54.53	6025	0.01	3.72
NWC PacBio						
Canu	44.27	62.25	33.51	—	0.00	36.51
Canu_Racon	44.32	25.32	**92.91**	—	0.00	36.51
Canu_HERO	44.24	27.46	34.27	—	0.00	36.51

Table S5. Assemble raw reads into contigs using Canu (first line), then polish the contigs respectively using Racon (second line) and HERO (third line). The table above shows the error rates of the unpolished contigs evaluated by QUAST, as well as the error rates of the contigs polished by Racon and HERO respectively. Indels/100 kbp: average number of insertion or deletion errors per 100,000 aligned bases. Mismatches/100 kbp = average number of mismatch errors per 100,000 aligned bases. Genome Fraction GF reflects how much of each of the strain-specific genomes is covered by the corrected reads. NGA50 is the length of the longest contig such that the alignments of that and all longer contigs span at least 50% of the reference sequence. N/100 kbp denotes the average number of uncalled bases (N's) per 100,000 bases in the read. MC = fraction of misassembled contigs.

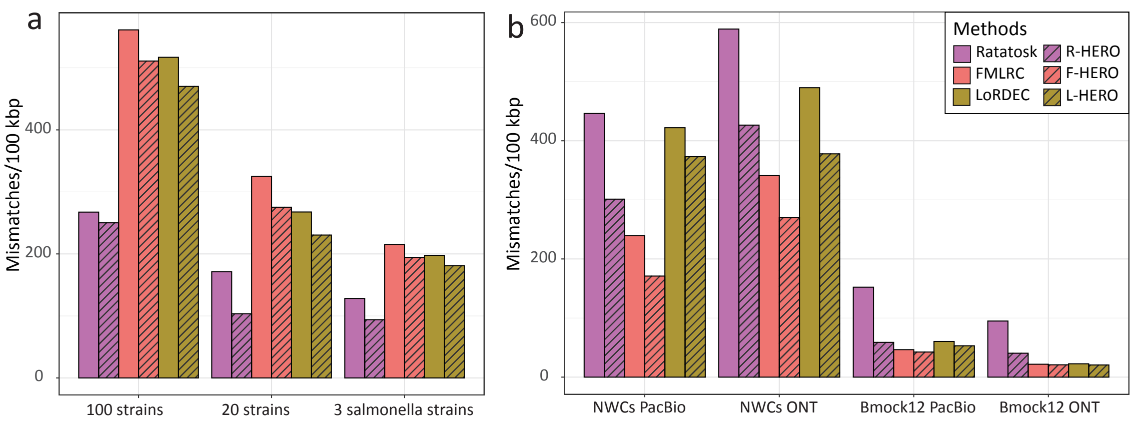

Figure S1. Mismatch error rates (y-axis) for simulated (a) and real data sets (b). The x-axis refers to different data sets and different protocols (different colors and patterns of bars). Ratatosk, FMLRC and LoRDEC refer to 8 iterations of the respective methods (pointed out as optimal protocol earlier); R-, F- and L-HERO refer to 3 iterations of Ratatosk, FMLRC and LoRDEC, respectively, followed by 5 iterations of HERO-OG.

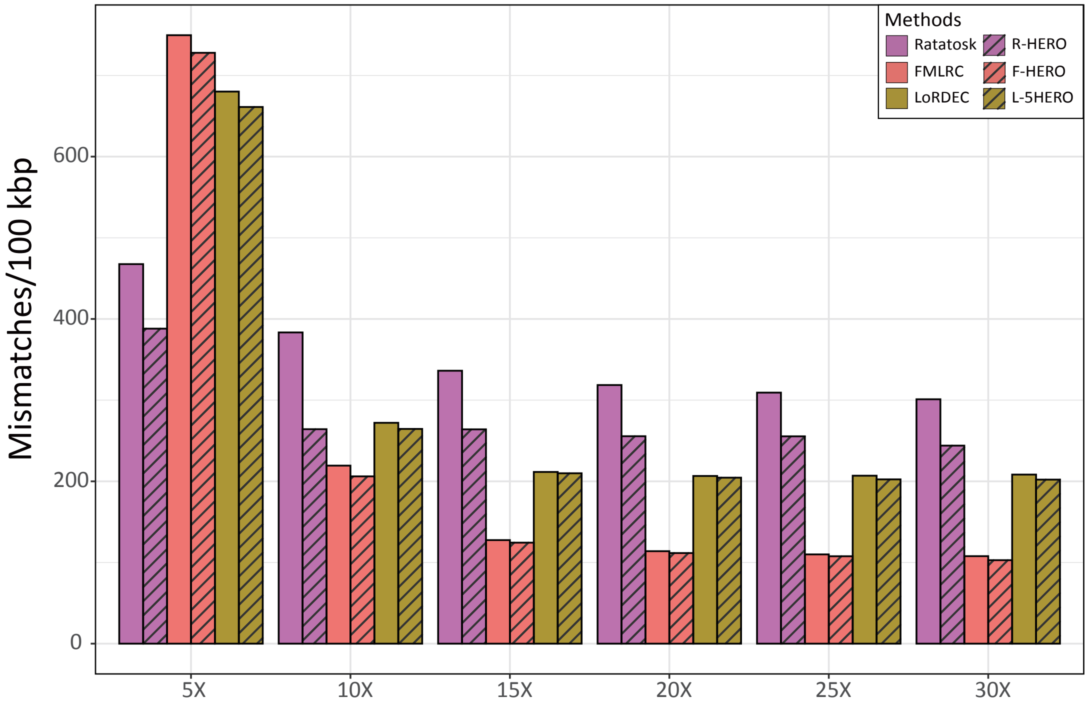

Figure S2. Mismatch error rates (y-axis) for spike-in datasets relative to different coverages (x-axis). Reads are from PacBio CLR. Different correction protocols are indicated by different colors and patterns of bars.

Strain ID	Coverage	Ratatosk	R-HERO	FMLRC	F-HERO	LoRDEC	L-HERO
NWCs PacBio indel error rate (per 100 kbp)							
CP029252.1	56.29X	135.47	61.90	111.87	38.08	38.09	28.25
CP031021.1	55.07X	140.38	65.21	107.32	39.02	39.55	29.89
CP029250.1	39.38X	315.90	207.91	270.4	133.89	69.6	45.59
CP031023.1	35.13X	525.85	344.70	316.34	174.11	88.35	53.18
CP031018.1	17.59X	1863.98	311.91	735.16	345.42	467.67	144.21
CP031016.1	10.27X	1241.70	601.76	720.18	386.42	361.26	208.04
NWCs PacBio mismatch error rate (per 100 kbp)							
CP029252.1	56.29X	129.67	85.86	80.78	41.31	146.83	129.00
CP031021.1	55.07X	139.12	94.56	85.89	50.14	161.33	143.43
CP029250.1	39.38X	340.70	266.84	200.70	141.96	353.30	292.81
CP031023.1	35.13X	609.09	507.34	298.45	242.30	489.01	447.12
CP031018.1	17.59X	2426.42	861.82	1686.14	990.91	2579.72	1025.03
CP031016.1	10.27X	1161.46	759.92	976.18	695.89	1013.44	806.4
Bmock12 PacBio indel error rate (per 100 kbp)							
2615840527	618.76X	307.58	39.63	9.74	5.62	24.77	11.70
2623620618	579.87X	302.67	54.44	28.51	17.96	33.36	15.92
2623620617	507.08X	335.53	64.08	32.57	18.45	36.27	18.41
2615840697	447.83X	319.17	51.73	19.65	12.18	33.09	16.28
2617270709	425.47X	366.38	47.86	14.85	8.81	38.32	19.26
2615840601	170.59X	368.56	87.83	29.66	20.63	26.84	14.58
2616644829	135.05X	326.39	75.08	23.59	16.98	38.77	19.23
2615840533	78.32X	288.00	37.19	51.49	39.82	38.19	16.61
2615840646	31.90X	392.87	46.28	129.88	100.46	173.40	55.15
2623620567	18.19X	558.84	93.40	216.20	173.68	182.99	57.48
2623620557	14.91X	648.11	146.43	203.76	167.54	231.07	64.72
Bmock12 PacBio mismatch error rate (per 100 kbp)							
2615840527	618.76X	97.97	15.67	7.61	6.32	14.54	11.13
2623620618	579.87X	207.90	107.03	60.92	58.18	79.66	76.71
2623620617	507.08X	216.57	108.61	87.81	83.38	85.49	79.13
2615840697	447.83X	135.68	41.05	37.95	34.49	67.06	59.67
2617270709	425.47X	117.16	29.52	17.99	16.28	39.74	30.05
2615840601	170.59X	117.00	40.43	24.15	21.94	20.03	20.10
2616644829	135.05X	167.61	84.43	59.20	58.27	74.14	72.09
2615840533	78.32X	95.66	17.57	51.36	45.52	24.80	19.79
2615840646	31.90X	123.86	22.50	117.03	99.49	131.15	96.56
2623620567	18.19X	281.03	98.04	308.89	301.86	268.87	218.36
2623620557	14.91X	328.49	143.96	330.53	325.88	267.24	180.00

Figure S3. Mismatch error rates for different methods stratified by the strains of the real data sets (1st column: NWC = Genbank ID, Bmock12 = IMG Taxon ID), which are ordered in descending order by their coverages (2nd column). Error rates are colored from large (red) via medium (white) to low (blue). The evident trend are improvements in indel error rate relative to increasing coverage.

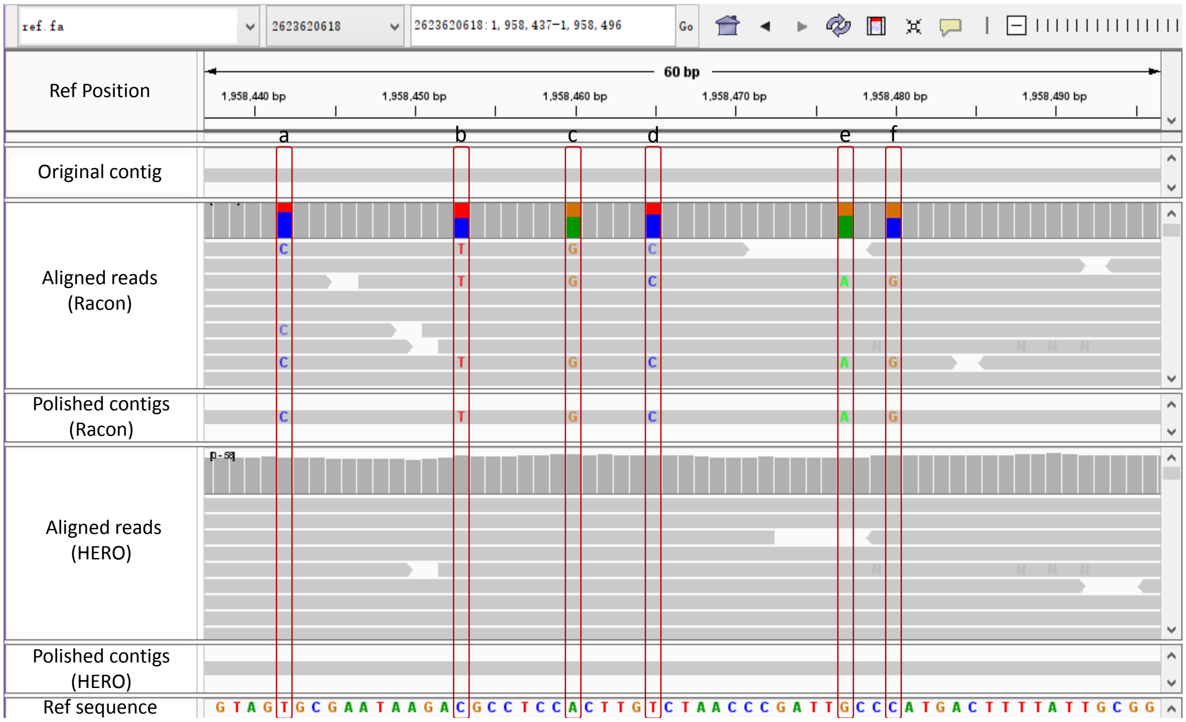

Figure S4. Taking Bmock12 PacBio data as an example to briefly describe over correction. Both Racon and HERO adopt the strategy of aligning reads to contigs. Here we can see that in Racon's alignment file, some reads have different bases compared to the original contigs. For example, at position a, most bases are C in Racon's alignment file but quite a few reads are T, which is the same as the original contigs. In HERO's alignment file, at position a, since there are more than 5 reads having the same base T as the original contigs, the C bases are considered as reads from other strains. HERO then disentangles the overlaps leaving only reads with T. They subsequently polish the contigs based on the alignment files. We can see Racon's polished contigs incorporated many bases different from the original contigs, such as at positions a, b, c, d, e, f. In contrast, HERO's polished contigs did not introduce any new mutations.
